# Supplementary material for: Causal Relationship between Aging and Anorexia Nervosa: A White-Matter-Microstructure-Mediated Mendelian Randomization Analysis
Source: Biomedicines. 2024 Aug 16;12(8):1874. doi: 10.3390/biomedicines12081874 (PMC11351342; doi:10.3390/biomedicines12081874)
Supplement: Supplementary file 1 [file biomedicines-12-01874-s001.zip › Supplementary Figure/Figure S4/Singe SNP plot of MR analysis for TL to ubm-b-1587.pdf]

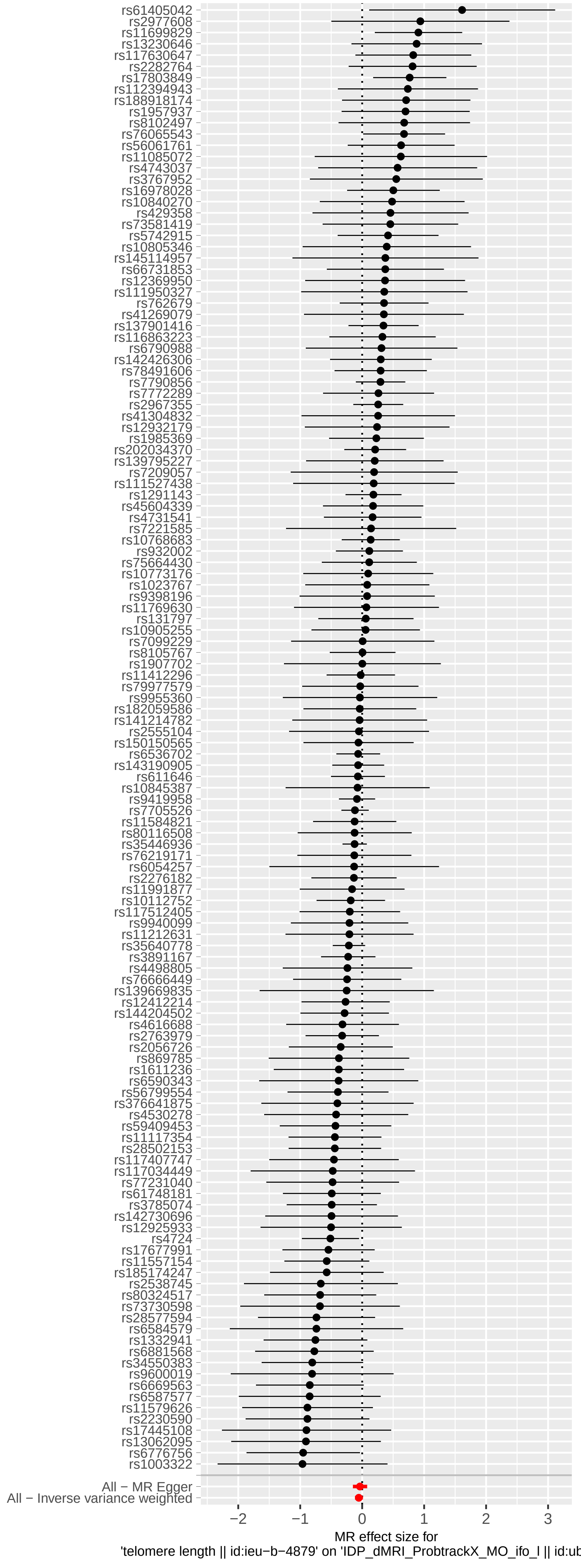
rs61405042
rs2977608
rs11699829
rs13230646
rs117630647
rs2282764
rs17803849
rs112394943
rs188918174
rs1957937
rs8102497
rs76065543
rs56061761
rs11085072
rs4743037
rs3767952
rs16978028
rs10840270
rs429358
rs73581419
rs5742915
rs10805346
rs145114957
rs66731853
rs12369950
rs111950327
rs762679
rs41269079
rs137901416
rs116863223
rs6790988
rs142426306
rs78491606
rs7790856
rs7772289
rs2967355
rs41304832
rs12932179
rs1985369
rs202034370
rs139795227
rs7209057
rs111527438
rs1291143
rs45604339
rs4731541
rs7221585
rs10768683
rs932002
rs75664430
rs10773176
rs1023767
rs9398196
rs11769630
rs131797
rs10905255
rs7099229
rs8105767
rs1907702
rs11412296
rs79977579
rs9955360
rs182059586
rs141214782
rs2555104
rs150150565
rs6536702
rs143190905
rs611646
rs10845387
rs9419958
rs7705526
rs11584821
rs80116508
rs35446936
rs76219171
rs6054257
rs2276182
rs11991877
rs10112752
rs117512405
rs9940099
rs11212631
rs35640778
rs3891167
rs4498805
rs76666449
rs139669835
rs12412214
rs144204502
rs4616688
rs2763979
rs2056726
rs869785
rs1611236
rs6590343
rs56799554
rs376641875
rs4530278
rs59409453
rs11117354
rs28502153
rs117407747
rs117034449
rs77231040
rs61748181
rs3785074
rs142730696
rs12925933
rs4724
rs17677991
rs11557154
rs185174247
rs2538745
rs80324517
rs73730598
rs28577594
rs6584579
rs1332941
rs6881568
rs34550383
rs9600019
rs6669563
rs6587577
rs11579626
rs2230590
rs17445108
rs13062095
rs6776756
rs1003322
All − MR Egger
All − Inverse variance weighted
−2
−1
0
1
2
3
MR effect size for
'telomere length || id:ieu−b−4879' on 'IDP_dMRI_ProbtrackX_MO_ifo_l || id:ub
